# Facts from Correspondents

**Published:** 1847-01

**Authors:** 


### FACTS FROM CORRESPONDENTS.

---

### TINCTURE OF MURIATE OF IRON IN GONNORRHŒA.

Dr. Osborne, of Erie, Alabama, informs us that he has continued to use the tincture of the muriate of iron beneficially in leucorrhœa, and that he has extended its application to some cases of gleet, and one of gonorrhœa, with success. The latter disease yielded to it almost immediately, as it is said to do to the nitrate of silver, the mode of applying which is noticed in another department of this number. Dr. Osborne uses the tincture of iron according to the following formula:

R. Tinct. mur. iron gtt. 10
Cold water ℥ ss.

Mix and inject with a syringe. In gonorrhœa he prescribes 15 drops of the tincture to the half ounce of water, to be employed as an injection three times daily.

### TINCTURE OF IODINE IN DROPSY.

The same correspondent, in a letter dated 30th November, mentions a case of ascites, resulting from peritonitis, in which he employed the tincture of iodine successfully, after the ordinary remedies for dropsy had been tried in vain, and the disease had progressed so far as to render tapping necessary. He promises a full report of the case. He does not state how he used the iodine—externally, however, we presume.

### FRACTURE OF THE SKULL; LOSS OF A PORTION OF THE BRAIN; RECOVERY.

The particulars of this interesting case have been communicated to us by Dr. Greenley, of Westpoint, Ky., but his letter reached us after the matter of this number was made up. It will appear in our February number.

